# Supplementary material for: The effects of postoperative treadmill exercise on rats with secondary lymphedema
Source: PLoS One. 2023 May 23;18(5):e0285384. doi: 10.1371/journal.pone.0285384 (PMC10204966; doi:10.1371/journal.pone.0285384)
Supplement: S1 Table — (DOCX) [file pone.0285384.s001.docx]

**Supplementary Table S1** Classification of ICG pattern in both groups.

| ICG lymphography pattern, n | | Week 1 | Week 3 | | Week 5 |
| --- | --- | --- | --- | --- | --- |
| Exercise Group (n=6) | |  |  | |  |
| Linear  Splash with linear  Stardust  Diffuse  None | | 0  1  0  2  3 | 2  1  2  1  0 | | 3  1  1  0  1 |
| Control Group (n=6) |  | |  |  | |
| Linear  Splash with linear  Stardust  Diffuse  None | 0  1  0  3  2 | | 0  1  3  2  0 | 2  3  1  0  0 | |

Data are presented as numbers.
