# Supplementary material for: The effects of postoperative treadmill exercise on rats with secondary lymphedema
Source: PLoS One. 2023 May 23;18(5):e0285384. doi: 10.1371/journal.pone.0285384 (PMC10204966; doi:10.1371/journal.pone.0285384)
Supplement: S1 Fig — (DOCX) [file pone.0285384.s002.docx]

**Supplementary FigureS1.**

**
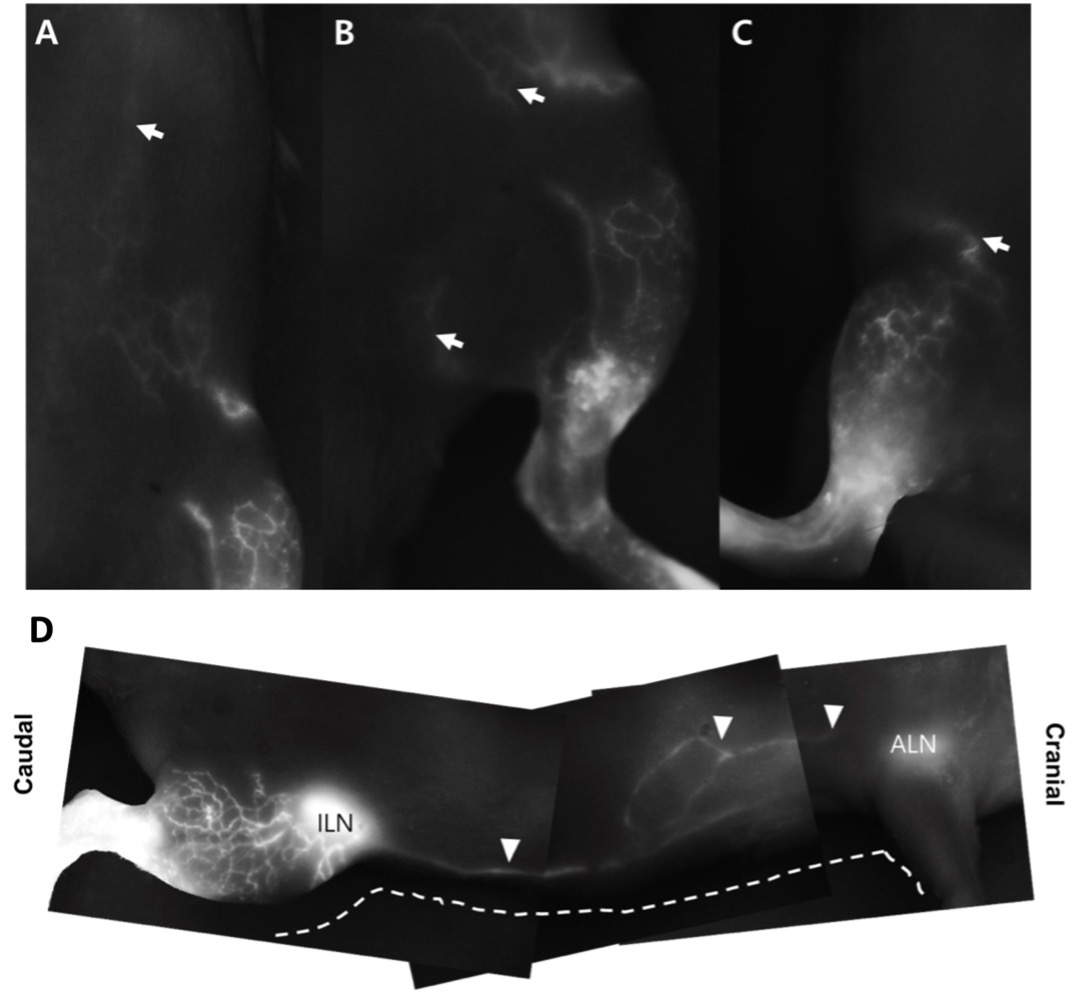
**

Visualized lymphatic pathways with ICG lymphography. (A-C) Representative ICG images of a rat in the exercise group (n = 1). New pathways were observed beyond the incision line in the groin region toward the axillary region (arrow) four weeks after beginning exercise. Ventral side abdominal area, thigh area, and lateral side thigh area, respectively. (D) Representative image of a rat that had undergone removal of popliteal LN on ventral side at week 4 (n = 3). The arrow heads indicate a thin lymph vessel connected with the ILN toward the ALN along the lateral intermodal vessel. ICG, indocyanine green; ILN, inguinal lymph node; ALN, axillary lymph node.
