# Supplementary material for: The effects of postoperative treadmill exercise on rats with secondary lymphedema
Source: PLoS One. 2023 May 23;18(5):e0285384. doi: 10.1371/journal.pone.0285384 (PMC10204966; doi:10.1371/journal.pone.0285384)
Supplement: Attachment — Submitted filename: Response to Reviewers.docx [file pone.0285384.s005.docx]

Shimpei Miyamoto, M.D., Ph.D.

Academic Editor

*PLOS ONE*

April 7th, 2023

Dear Dr. Miyamoto:

On behalf of the authors, I would like to thank you and the reviewers for the valuable and helpful comments on our submitted manuscript, “The effects of postoperative treadmill exercise on rats with secondary lymphedema” (PONE-D-22-30319).

We have carefully reviewed the comments and have revised the manuscript accordingly. Our responses are provided in a point-by-point manner, and changes to the manuscript are shown in yellow highlight, which are also visible through the Track Changes function.

We would be happy to make further corrections if necessary, and look forward to hearing from you soon.

Sincerely,

Jae Yong Jeon, M.D., Ph.D.

Department of Rehabilitation Medicine

Asan Medical Center, University of Ulsan College of Medicine

88, Olympic-ro 43-gil, Songpa-gu, Seoul 05505, Republic of Korea

Tel: 82-2-3010-3791	Fax: +82-2-3010-6964

E-mail: [jyjeon71@gmail.com](mailto:jyjeon71@gmail.com)

**Response to Reviewer 2**

Thank you for reviewing our manuscript. Our answers to your queries are as follows.

**Comment 1**: I still would like to receive a clear answer regarding the following point:

“Do you have a baseline of the ICG study to support its use in your model?” The Reviewers and the readers know the staging of lymphedema according to ICG findings in humans. And this is not applicable to other models unless validated. As this imaging technique has not been extensively performed on animal models, authors are encouraged to have a baseline to compare with postoperative findings.

**Response:** Thank you for bringing this to our attention. Previous studies have utilized rats in order to observe lymphatic drainage patterns via ICG lymphography. Ogata et al. analyzed ICG images of hindlimbs from rats that had undergone lymph node dissection seven days after surgery. Their results indicated that the ICG images displayed diffuse or blurry patterns. Additionally, Takeno et al. reported that in a hindlimb non-operative rat model, a linear signal was observed, while in the operative hindlimb after inguinal and popliteal lymph node dissection, dermal backflow patterns, such as stardust, were shown. This suggests that detecting the linear pattern earlier in the operated limb will result in a quicker recovery process and a return to the state prior to surgery. We have described these findings on page 12, lines 249 to 254 in the main text.

Please refer to the list of previous studies I mentioned below:

1. Ogata F, Azuma R, Kikuchi M, Koshima I, Morimoto Y. Novel lymphography using indocyanine green dye for near-infrared fluorescence labeling. Ann Plast Surg. 2007 Jun;58(6):652-5. doi: 10.1097/01.sap.0000250896.42800.a2. PMID: 17522489.
2. Takeno Y, Fujimoto E. Alterations of lymph flow after lymphadenectomy in rats revealed by real time fluorescence imaging system. Lymphology. 2013 Mar;46(1):12-9. PMID: 23930437.

**Additional clarifications**

In addition to the above comments, all spelling and grammatical errors have been corrected. Furthermore, references 3 (page 15, lines 316 – 318) and 7 (page 16, lines 331 – 332) have been updated with the latest information.

Thank you again for reviewing our manuscript in detail and providing helpful comments. We hope that our responses and the corresponding revisions are satisfactory.
